# Supplementary material for: Evaluating factors contributing to the failure of information system in the banking industry
Source: PLoS One. 2022 Mar 17;17(3):e0265674. doi: 10.1371/journal.pone.0265674 (PMC8929651; doi:10.1371/journal.pone.0265674)
Supplement: S1 File — (DOCX) [file pone.0265674.s002.docx]

**Appendix A**

Basic equations of rough set theory and rough number [43] are given below.

Considering there are $n$ classes of experts’ opinion,$R=\{C1,C2,\ldots,Cn\}$, which are in the order $C1<C2<\cdots<Cn$, and $Y$ is an arbitrary object of $U$, then the upper and lower approximations of $C_{i}$ and the boundary region are evaluated by,

Lower approximation:

| $\underline{Apr}\left( C_{i} \right)=U\left\{ Y\in U/R\left( Y \right)\leq C_{i} \right\}$ | (1) |
| --- | --- |

Upper approximation:

| $\bar{Apr}\left( C_{i} \right)=U\left\{ Y\in U/R\left( Y \right)\geq C_{i} \right\}$ | (2) |
| --- | --- |

Boundary region:

| $Bnd\left( C_{i} \right)=U\left\{ Y\in U/R\left( Y \right)\neq C_{i} \right\}$ | | |
| --- | --- | --- |
|  | $=\left\{ Y\in U/R\left( Y \right)>C_{i} \right\}\cup\left\{ Y\in U/R\left( Y \right)<C_{i} \right\}$ | (3) |

Hence, the class $C_{i}$ can be represented in the form of a rough number, which contains the lower limit $\underline{Lim}\left( C_{i} \right)$ and upper limit $\bar{Lim}\left( C_{i} \right)$ and can be calculated as,

| $\underline{Lim}\left( C_{i} \right)=\frac{1}{N_{L}}\sum R\left( Y \right)\vert Y\in\underline{Apr}\left( C_{i} \right)$ | (4) |
| --- | --- |
| $\bar{Lim}\left( C_{i} \right)=\frac{1}{N_{U}}\sum R\left( Y \right)\vert Y\in\bar{Apr}\left( C_{i} \right)$ | (5) |

where, $N_{L}$ represents the number of objects included for lower approximation of $C_{i}$, and $N_{U}$ is the number of objects included for the upper approximation of $C_{i}$.

The experts’ subjective decisions can be expressed in terms of rough interval form on the basis of lower limit $\underline{Lim}\left( C_{i} \right)$ and upper limit $\bar{Lim}\left( C_{i} \right)$.

Rough number:

| $RN\left( C_{i} \right)=\left[ \overline{Lim}\left( C_{i} \right), \underline{Lim}\left( C_{i} \right) \right]$ | (6) |
| --- | --- |

The degree of accuracy of decisions by decision-makers can be analyzed by finding the interval of the boundary region, and the smaller the interval of a rough number, the greater the precision is.

The interval of boundary region:

| $IBR\left( C_{i} \right)=\overline{Lim}\left( C_{i} \right)-\underline{Lim}\left( C_{i} \right)$ | (7) |
| --- | --- |

The arithmetic operations for rough numbers are done as follows:

Addition of rough numbers ${RN}_{1}$ and ${RN}_{2}$,

| ${RN}_{1}+{RN}_{2}=\left( \underline{Lim}_{1}, \overline{Lim}_{1} \right)+\left( \underline{Lim}_{2}, \overline{Lim}_{2} \right)=\left( \underline{Lim}_{1}+\underline{Lim}_{2},\overline{Lim}_{1}+\overline{Lim}_{2} \right)$ | (8) |
| --- | --- |

Subtraction of rough numbers ${RN}_{1}$ and ${RN}_{2}$,

| ${RN}_{1}-{RN}_{2}=\left( \underline{Lim}_{1}, \overline{Lim}_{1} \right)-\left( \underline{Lim}_{2}, \overline{Lim}_{2} \right)=\left( \underline{Lim}_{1}-\underline{Lim}_{2},\overline{Lim}_{1}-\overline{Lim}_{2} \right)$ | (9) |
| --- | --- |

Multiplication of rough numbers ${RN}_{1}$ and ${RN}_{2}$,

| ${RN}_{1}\times{RN}_{2}=\left( \underline{Lim}_{1}, \overline{Lim}_{1} \right)\times\left( \underline{Lim}_{2}, \overline{Lim}_{2} \right)=\left( \underline{Lim}_{1}\times\underline{Lim}_{2},\overline{Lim}_{1}\times\overline{Lim}_{2} \right)$ | (10) |
| --- | --- |

Division of rough numbers ${RN}_{1}$ and ${RN}_{2}$,

| ${RN}_{1}\div{RN}_{2}=\left( \underline{Lim}_{1}, \overline{Lim}_{1} \right)\div\left( \underline{Lim}_{2}, \overline{Lim}_{2} \right)=\left( \underline{Lim}_{1}\div\overline{Lim}_{2}, \underline{Lim}_{2}\div\overline{Lim}_{1} \right)$ | (11) |
| --- | --- |

Scalar multiplication of rough number ${RN}_{1}$ with non-zero constant $k$,

| $k\times{RN}_{1}={k\times\underline{Lim}}_{1}, k\times\overline{Lim}_{1}$ | (12) |
| --- | --- |

**Appendix B**

**The questionnaire for the survey is presented as follows:**

Rating Method: Ratings are provided on a priority basis. The higher the rating the more important the impact is. Score 1 means almost unimportant, while score 10 indicates absolutely important, other scores are also defined similarly. Several criteria can be marked by the same rating. Rate the following criteria on a scale of 1-10.

At first, the evaluating criteria are rated based upon the opinion of experts.

| **Criteria** | **Rating (1-10)** |
| --- | --- |
| Severity- [importance ifFailure turns to the severe situation] |  |
| Occurrence- [importance ifFailure takes place frequently] |  |
| Detection Difficulty- [importance ifFailure is difficult to detect] |  |
| Time- [importance of Time duration for detection &Solution] |  |
| Cost- [importance of Cost incurred to solve the problem] |  |

Failure modes are then rated based on different criteria based upon the opinion of experts (rated from 1-10).

| **Failure Mode** | **Severity** | **Occurrence** | **Detection Difficulty** | **Time** | **Cost** |
| --- | --- | --- | --- | --- | --- |
| Data-Base Hack |  |  |  |  |  |
| Server failure |  |  |  |  |  |
| Virus Effect |  |  |  |  |  |
| Cipher to Plain Text Malfunction |  |  |  |  |  |
| Character Misspelled |  |  |  |  |  |
| Wrong Message Transcription |  |  |  |  |  |
| Peripheral Error |  |  |  |  |  |
| Broadcast Data Missing  (Up/Down) link failure |  |  |  |  |  |
| Cyber Attack |  |  |  |  |  |
| Third Party intervention |  |  |  |  |  |
| Network interruption |  |  |  |  |  |

**Sample feedback obtained from Expert no. 9**

**Failure factors in the IT system of the banking industry**

Rating Method: Ratings are provided on a priority basis. The higher the rating the more important the impact is. Score 1 means almost unimportant, while score 10 indicates absolutely important, other scores are defined similarly. Several criteria can be marked with the same rating. Rate the following criteria on a scale of 1-10.

At first, the evaluating criteria are rated based upon the opinion of experts.

| **Criteria** | **Rating (1-10)** |
| --- | --- |
| Severity- [importance ifFailure turns to severe situation] | 5 |
| Occurrence- [importance ifFailure takes place frequently] | 9 |
| Detection Difficulty- [importance ifFailure is difficult to detect] | 10 |
| Time- [importance ofTime duration for detection & Solution] | 9 |
| Cost- [importance ofCost incurred to solve the problem] | 9 |

Failure modes are then rated on the basis of different criteria based upon the opinion of experts. (rated from 1-10)

| **Failure Mode** | **Severity** | **Occurrence** | **Detection Difficulty** | **Time** | **Cost** |
| --- | --- | --- | --- | --- | --- |
| Data-Base Hack | 10 | 9 | 8 | 8 | 10 |
| Server failure | 9 | 9 | 9 | 9 | 10 |
| Virus Effect | 8 | 8 | 10 | 10 | 10 |
| Cipher to Plain Text Malfunction | 2 | 2 | 2 | 2 | 2 |
| Character Misspelled | 1 | 1 | 1 | 1 | 1 |
| Wrong Message Transcription | 1 | 1 | 1 | 1 | 1 |
| Peripheral Error | 1 | 1 | 1 | 1 | 1 |
| Broadcast Data Missing  (Up/Down) link failure | 1 | 1 | 1 | 1 | 1 |
| Cyber Attack | 10 | 9 | 10 | 10 | 10 |
| Third Party intervention | 5 | 5 | 4 | 5 | 6 |
| Network interruption | 9 | 9 | 10 | 10 | 10 |

**Sample feedback obtained from Expert no. 15**

**Failure factors in the IT system of the banking industry**

Rating Method: Ratings are provided on a priority basis. The higher the rating the more important the impact is. Score 1 means almost unimportant, while score 10 indicates absolutely important, other scores are defined similarly. Several criteria can be marked with the same rating. Rate the following criteria on a scale of 1-10.

At first, the evaluating criteria are rated based upon the opinion of experts.

| **Criteria** | **Rating (1-10)** |
| --- | --- |
| Severity- [importance ifFailure turns to severe situation] | 10 |
| Occurrence- [importance ifFailure takes place frequently] | 8 |
| Detection Difficulty- [importance ifFailure is difficult to detect] | 7 |
| Time- [importance ofTime duration for detection & Solution] | 8 |
| Cost- [importance ofCost incurred to solve the problem] | 6 |

Failure modes are then rated on the basis of different criteria based upon the opinion of experts. (rated from 1-10)

| **Failure Mode** | **Severity** | **Occurrence** | **Detection Difficulty** | **Time** | **Cost** |
| --- | --- | --- | --- | --- | --- |
| Data-Base Hack | 10 | 10 | 9 | 10 | 5 |
| Server failure | 9 | 9 | 9 | 9 | 4 |
| Virus Effect | 8 | 7 | 8 | 8 | 3 |
| Cipher to Plain Text Malfunction | 8 | 8 | 6 | 6 | 8 |
| Character Misspelled | 5 | 4 | 3 | 5 | 7 |
| Wrong Message Transcription | 6 | 6 | 5 | 6 | 7 |
| Peripheral Error | 5 | 7 | 8 | 6 | 5 |
| Broadcast Data Missing  (Up/Down) link failure | 7 | 8 | 8 | 9 | 7 |
| Cyber Attack | 8 | 8 | 7 | 8 | 8 |
| Third Party intervention | 6 | 5 | 3 | 5 | 6 |
| Network interruption | 8 | 9 | 8 | 9 | 8 |
